# New Cholinesterase Inhibitory Constituents from *Lonicera quinquelocularis*

**DOI:** 10.1371/journal.pone.0094952

**Published:** 2014-04-14

**Authors:** Dilfaraz Khan, Hidayat Ullah Khan, Farmanullah Khan, Shafiullah Khan, Syed Badshah, Abdul Samad Khan, Abdul Samad, Farman Ali, Ihsanullah Khan, Nawshad Muhammad

**Affiliations:** 1 Department of Chemistry, Gomal University, Dera Ismail Khan, Khyber Pakhtunkhwa, Pakistan; 2 Departmet of Biotechnology, University of Science and Technology, Bannu, Khyber Pakhtunkhwa, Pakistan; 3 Department of Chemistry, University of Science and Technology, Bannu, Khyber Pakhtunkhwa, Pakistan; 4 Interdisciplinary Research Center in Biomedical Materials, COMSATS Institute of Information Technology, Lahore, Pakistan; 5 Department of Chemistry, Shaheed BBB University, Sheringal, Dir, Khyber Pakhtunkhwa, Pakistan; 6 Department of Fundamental and Applied Sciences, Centre of Ionic Liquid, University of Technology Petronas, Perak, Malaysia; Weizmann Institute of Science, Israel

## Abstract

A phytochemical investigation on the ethyl acetate soluble fraction of *Lonicera quinquelocularis* (whole plant) led to the first time isolation of one new phthalate; *bis(7-acetoxy-2-ethyl-5-methylheptyl) phthalate* (**3**) and two new benzoates; *neopentyl-4-ethoxy-3, 5-bis (3-methyl-2-butenyl benzoate* (**4**) *and neopentyl-4-hydroxy-3, 5-bis (3-methyl-2-butenyl benzoate* (**5**) along with two known compounds *bis (2-ethylhexyl phthalate* (**1**) and *dioctyl phthalate* (**2**). Their structures were established on the basis of spectroscopic analysis and by comparison with available data in the literature. All the compounds (**1–5**) were tested for their acetylcholinesterase (AChE) and butyrylcholinesterase (BChE) inhibitory activities in dose dependent manner. The IC_50_ (50% inhibitory effect) values of compounds **3** and **5** against AChE were 1.65 and 3.43 µM while the values obtained against BChE were 5.98 and 9.84 µM respectively. Compounds **2** and **4** showed weak inhibition profile.

## Introduction

**Figure 1:**
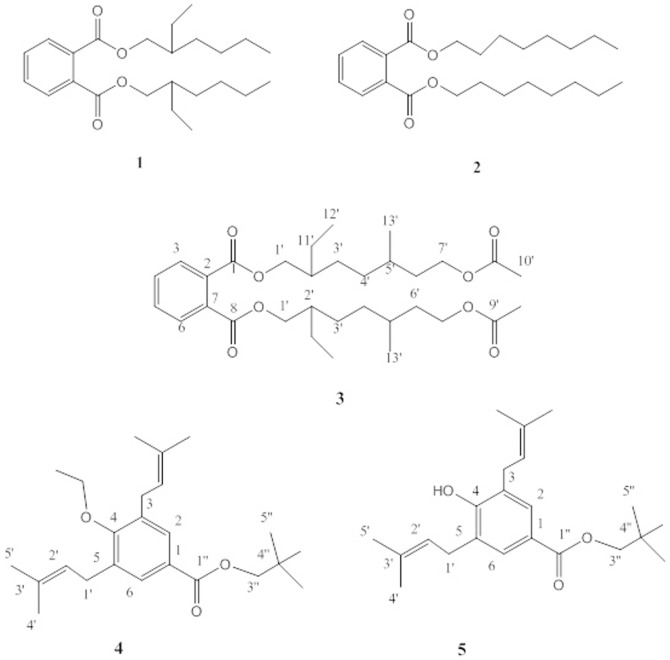
Structures of compounds 1–5 from *L. quinquelocularis*.

The genus Lonicera belongs to the family *Caprifoliaceae* which consists of 12 genera and 450 species mainly present in the temperate region of Northern Hemisphere [1]. In Pakistan, the genus Lonicera has 4 genera and 27 species [2]. Various species of this genus have been used for the treatment of acute fever, headache, respiratory infections [3], antibacterial [4], antioxidant [5], [6], cytoprotective [7], hepatoprotective [8], [9], antiviral [10], antitumor [11], [12] and anti-inflamatory activities [13]. Previous studies on this genus reported the isolation of a variety of constituents including iridoids, bisiridoids, sulfur containing monoterpenoids, alkaloidal glycosides, triterpenoids, saponins, coumarin glycosides and flavone glycosides [14]–[17]. *Lonicera quinquelocularis* is a member of this genus which is widely distributed in dry sunny places of Asia with altitudes of 750–3000 meters. In Pakistan, it is present in Baluchistan, Kurram, Chitral, Swat, Astor, Hazara, Murree hills, Poonch and Kashmir [18]. Previous phytochemical study on this plant reported the isolation of triterpenoid, benzoates, lonicerin, loganin, coumarin and iridoide glycosides [6], [19]. It has been widely used as antipyretic and antioxidant and in the treatment of hypotension, sedation etc. [20], [21]. The diverse medicinal importance of genus Lonicera has prompted us to investigate the phytochemical constituents of *L. quinquelocularis*. In this study the isolation and structural elucidation of two known compounds; *bis (2-ethylhexyl phthalate* (**1**), *dioctyl phthalate* (**2**) followed by three new compounds; *bis(7-acetoxy-2-ethyl-5-methylheptyl phthalate* (**3**), *neopentyl-4-ethoxy-3,5-bis (3-methyl-2-butenyl benzoate* (**4**) and *neopentyl-4-hydroxy-3,5-bis (3-methyl-2-butenyl benzoate* (**5**) respectively are reported (Fig. 1). The compounds **3** and **5** showed significant inhibitory activities against acetylcholinesterase (AChE) and butyrylcholinesterase (BChE) in a dose dependent manner.

## Results and Discussion

In the present investigation, phytochemical study was performed on the ethyl acetate extract of whole plant of *Lonicera quiquelocularis* afforded compounds **1** (15 mg, 0.11%), **2** (10 mg, 0.07%) and **3** (12 mg, 0.09%), **4** (21 mg, 0.16%) and **5** (19 mg, 0.14%). The known compounds **1** and **2** were isolated as colourless oils and were identified as *bis (2-ethylhexyl phthalate* and *dioctyl phthalate* by using spectroscopic analysis (Fig. S1a–S1d and S2a–S2d in File S1) [22]–[24].

The compound **3** was obtained as colourless oil and the IR spectrum of this compound showed strong absorption bands at 1725 cm^−1^ (ester carbonyl), 1610 cm^−1^ (aromatic) and 1130–1200 cm^−1^ (ether C–O). The UV bands were observed at 232 and 296 nm corresponding to the unsaturated carbonyl and aromatic ring respectively. The HR-EIMS (Fig. S3e in File S1) analysis showed molecular ion peak at *m/z* 562.7642 which was corresponded in establishing the formula C_32_H_50_O_8_ (formula mass 562.3603) with diagnostic ions at *m/z* 444 [M–C_4_H_6_O_4_], 387 [M– C_8_H_14_O_4_] and 277 [M– C_16_H_30_O_4_]. The ^1^H-NMR spectrum (Fig. S3a in File S1) showed two signals for aryl protons which were attributed to H-3/H6 (δ 7.73, 2H, dd, *J* = 5.8, 3.3 Hz) and H-4/H-5 (δ 7.55, 2H, dd, *J* = 5.8, 3.3 Hz). The 1-6 disubstituted aromatic ring was confirmed by A_2_B_2_ spin system of the aryl protons. A little downfield signals were found resonated at δ 4.15 (2H, dd, *J* = 11.8, 6.1 Hz) and 4.25 (2H, dd, *J* = 12.1, 6.4 Hz) corresponding to oxomethylenic protons H-1'a and H-1'b of the ester moiety of phthalate. The downfield signals were observed at δ 4.09 (4H, t, *J* = 6.4 Hz) which were a clear characteristic of another oxomethylenic protons H-7'. A singlet at δ 2.07 (6H, s, H-10') was assigned to methyl group attached to carbonyl function. The COSY (H–H) correlations (Fig. S3c in File S1) were seen to show pronounce vicinal correlation between H-1a' and H-1b' which confirmed the branched nature of the phthalate. The triplet for H-7' showed that there was no such branching near to correlate with it. In addition the HMBC clearly correlated the H-1' to the ester moiety on one side and H-7' with the ester moiety on the other side.

The ^13^C NMR spectrum (Fig. S3b in File S1) of compound **3** showed presence of 18 carbon atoms while their multiplicity was determined by DEPT experiment which indicated three methyl, six methylene, four methine and three quaternary carbons. The two carbonyl carbons resonated at δ 171.22 (C-1) and 167.75 (C-9') respectively. The aromatic carbons displayed signals at δ 132.49 (C-2, C-7), 130.86 (C-4, C-5) and 128.0 (C-3) while the oxomethylenic carbons (C-1' and C-7') were observed at δ 68.15 and 64.35 respectively. The methine carbon chain (C-2' and C-5') showed signals at δ 38.76 and 34.67 respectively, while methylenic carbons resonated at δ 31.60, 28.94, 30.39 and 23.76 (C-3', 4', 6' and 11') respectively. The methyl carbon of acyl group appeared at δ 22.65. The two methyl carbons were observed at δ 10.95 and 14.09 (Table 1)

**Table 1:** ^1^H and ^13^C NMR data and important HMBC correlations of compound **3** (CDCl_3_, δ in ppm, *J* in Hz).

H/C	^1^H NMR	^13^C NMR	HMBC
1	Q	171.22	_
2	Q	132.49	_
3 (CH)	7.73 (dd; 5.8, 3.3)	128.80	1, 2, 4, 5
4 (CH)	7.56 (dd; 5.8, 3.3)	130.86	2, 5, 6
5 (CH)	7.56 (dd; 5.8, 3.3)	130.86	4, 6, 7
6 (CH)	7.73 (dd; 5.8, 3.3)	128.80	4, 5, 7, 8
7	Q	132.49	_
8	Q	171.22	_
1' (CH_2_)	4.15 (dd; 11.8, 6.1, H-1'a), 4.25 (dd; 12.1, 6.4, H-1'b)	68.15	2', 11'
2' (CH)	1.70 (m)	38.76	1', 3', 11'
3' (CH_2_)	1.29 (m)	31.60	2', 11'
4' (CH_2_)	1.35 (m)	28.94	2', 5', 13'
5' (CH)	1.64 (m)	34.67	7', 13'
6' (CH_2_)	1.48 (m, H-6'b),1.32 (m, H-6'a)	30.39	5', 13'
7' (CH_2_)	δ 4.09 (t; 6.4)	64.35	5', 9'
9'	Q	167.75	_
10'(CH_3_)	δ 2.07 (s)	22.65	_
11' (CH)	1.45 (m)	23.76	9'
12' (CH_3_)	0.89 (t; 6.5)	10.95	1', 2', 3'
13' (CH_3_)	0.96 (d; 6.4)	14.09	_

**Figure 2:**
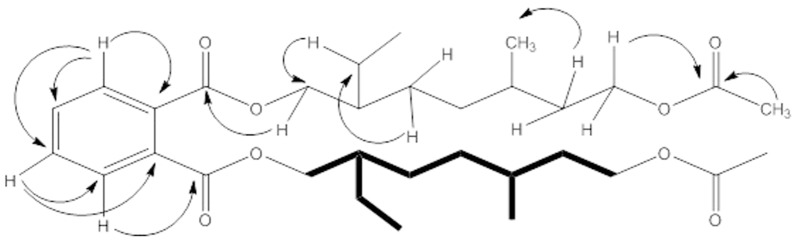
HMBC (H–C) correlations in compound 3.

The HMBC correlations (Fig. 2 and S3d in File S1) were in conformation with the assigned structure of compound **3**. The H-1' protons showed *^3^J* correlation with C-1 (171.22) and *^2^J* correlation with C-2' (38.76) and C-11' (23.76). H-7' was observed to have *^3^J* correlation with C-9' (167.75) and *^2^J* correlation with C-6' (30.39). Similarly *^2^J* correlation was observed between H-10' and C-9' of carbonyl carbon. The carbons and protons (^1^H/^13^C) connectivity along with some important HMBC correlations of compound **3** are shown in Table 1. On the basis of these evidences the structure of compound **3** was assigned as *bis (7-acetoxy-2-ethyl-5-methylheptyl phthalate*.

**Figure 3:**
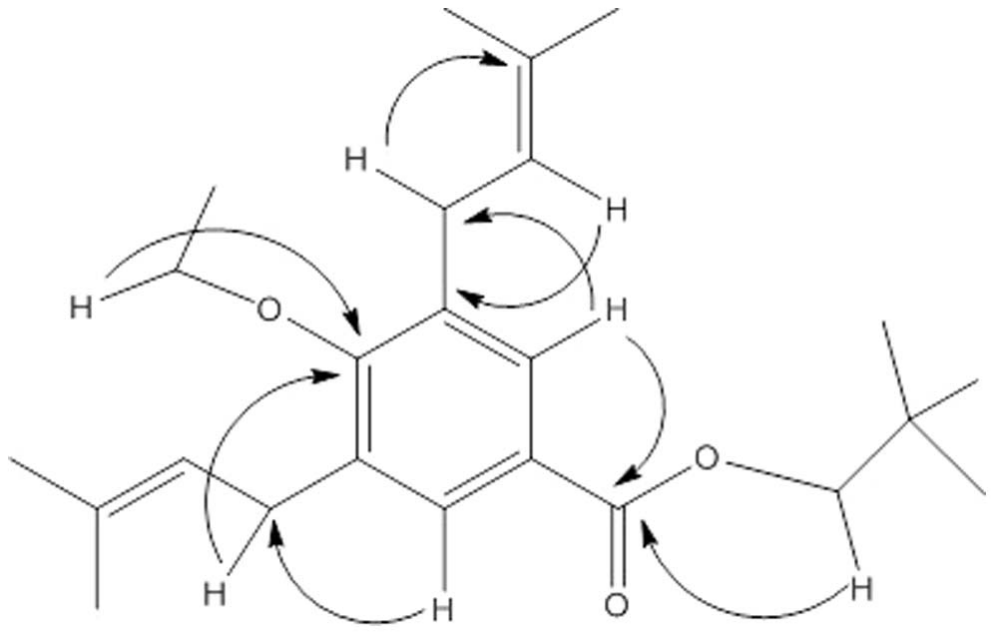
Important HMBC correlations in 4 (H→C).

The compound **4** was isolated as amorphous powder and its molecular formula was established as C_24_H_36_O_3_ by HR-EIMS analysis (Fig. S4c in File S1) corresponding to the molecular weight 372.3742 (calcd. 372.2156). The IR spectrum indicated the presence of ester (1705 cm^−1^), aromatic (1600–780 cm^−1^) and ether (1230 cm^−1^) functionalities in the structure. The ^1^H-NMR spectrum (Fig. S4a in File S1) displayed signals for two equivalent aromatic protons at *δ* 7.39 (s, *J* = 1.8 Hz), resonated at same chemical shift value confirming the 1,3,4,5 substituted aromatic ring. The signals observed at δ 5.69 (2H, t, *J* = 6.8 Hz, H-2'), δ 3.27 (4H, d, *J* = 6.8 Hz, H-1'), δ 1.83 (6H, s, H-4') and δ 1.74 (6H, s, H-5') suggested a side isoprene chain. The signal found at *δ* 4.15 (s, 2H) and δ 0.96 (s, 9H) was corresponded to the oxomethylene and methyl protons of neopentyl group of ester moiety. A quartet at δ 4.38 (2H, q, *J* = 7.8 Hz) and a triplet at δ 1.36 (3H, t, *J* = 7.8 Hz) were assigned to ethoxy group attached to the aromatic ring. The ^13^C-NMR (Fig. S4b in File S1) along with DEPT spectrum showed presence of 15 signals corresponding to four aromatic carbon atoms resonated at δ 154.6 (C-4), 137.2 (C-1), 129.3 (C-2, C-6), 124.8 (C-3, C-5) and two ethoxy carbons at *δ* 68.3 and 15.5 respectively. Five isoprene carbons displayed signals at δ 133.5 (C-3'), 121.5 (C-2'), 27.6 (C-1'), 24.7 (C-5') and 19.4 (C-4') and three signals for neopentyl group of ester moiety were observed at δ 83.2 (C-3''), 29.4 (C-4'') and 23.1 (C-5''). The ester carbonyl carbon was observed at δ 163.0. The assignments of the ^1^H and ^13^C NMR data were supported by 2D experiments (Fig. 3) and by comparing the data with the known derivatives of related benzoates [25]. In HMBC spectrum of compound **4** (Fig. S4d in File S1), H-2 and H-6 (*δ* 7.39) protons showed *^3^J*_H-C_ correlations with ester carbonyl carbon C-1'' (*δ* 168.2). Similarly *^3^J*_H-C_ correlations were found between H-2' (*δ* 5.69) and C-5, C-3 (*δ* 124.8) and between H-1' (*δ* 3.27) and C-2, C-6 (*δ* 129.3) confirming the positions of two isoprene side chains on the aromatic ring. The carbons and protons (^1^H/^13^C) connectivity along key HMBC correlations is provided in Table 2 which ultimately established the structure of compound **4** as *neopentyl-4-ethoxy-3, 5-bis (3-methyl-2-butenyl benzoate*.

**Table 2:** ^1^H and ^13^C NMR data (CDCl_3_) and ^1^H/^13^C correlations of compounds **4** and **5** (δ in ppm, *J* in Hz).

Compound 4			Compound 5		
H/C	^1^H NMR	^13^C NMR	^1^H NMR	^13^C NMR	HMBC
1	Q	137. 2	Q	135.0	
2 (CH)	7.39 (d; 1.8)	129.3	7.48 (d; 1.6)	129.3	C-4,C- 6, C-1', C-1''
3	Q	124.8	Q	124.2	_
4	Q	154.6	Q	152.5	_
5	Q	124.8	Q	124.2	_
6 (CH)	7.39 (d; 1.8)	129.3	7.48 (d; 1.6)	129.3	C-4, C-2, C-1', C-1''
1' (CH_2_)	3.27 (d; 6.8)	27.6	3.33 (d; 7.1)	27.3	C-2, C-6, C-4, C-3'
2' (CH)	5.69 (t; 6.8)	121.5	5.72 (t; 7.1)	123.1	C-3, C-5, C-4', C-5'
3'	Q	133.5	Q	133.5	_
4' (CH_3_)	1.83 (s)	19.4	1.85 (s)	19.1	C-2'
5' (CH_3_)	1.78 (s)	24.7	1.79 (s)	25.4	C-2'
1''	Q	163.0	Q	165.4	_
3'' (CH_2_)	4.15 (s)	83.2	4.19 (s)	81.9	C-1''
4''	Q	29.4	Q	29.7	_
5'' (CH_3_)	0.96 (s)	23.1	0.98 (s)	21.8	C-3''
OCH_2_–	4.38 (q; 7.8)	68.3	_	_	C-4
O–C–CH_3_	1.36 (t; 7.8)	15.5	_	_	_
OH	_	_	5.31 (s)	_	_

**Figure 4:**
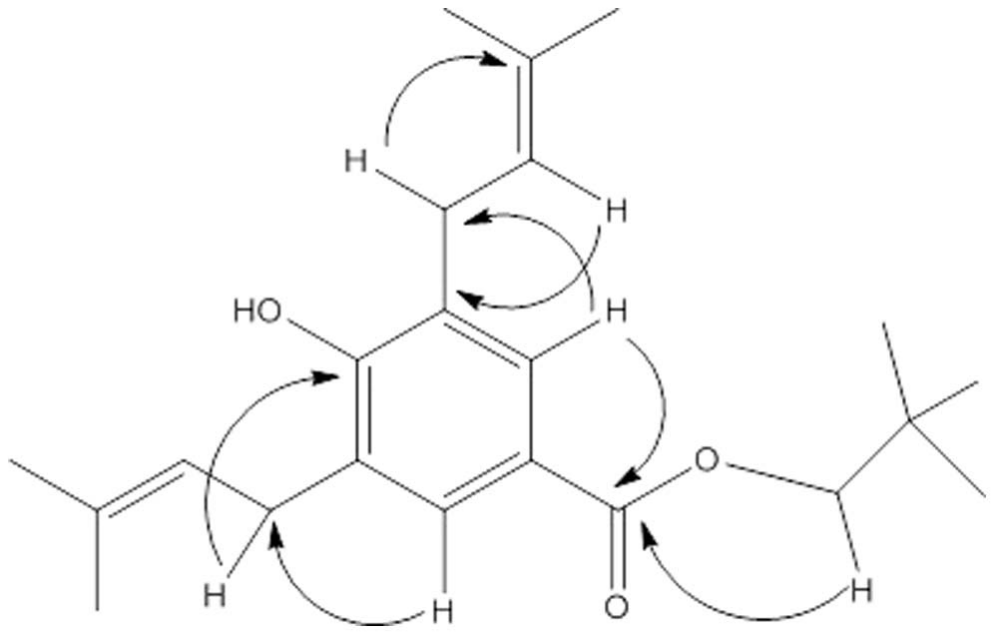
Important HMBC correlations in 5 (H→C).

The compound **5** was isolated as amorphous white solid and from its HR-EIMS analysis (Fig. S5c in File S1), a parent ion peak at *m/z* 344.6403 (calcd. 344.2549) was observed which suggested that the molecular formula would be C_22_H_32_O_3_. The spectrum of IR was indicating the presence of hydroxyl (3390 cm^−1^), ester (1725 cm^−1^) and aromatic (1600, 780 cm^−1^) functional groups. The ^1^H and ^13^C-NMR spectrum (Fig. S5a and S5b in File S1) of compound **5** showed same chemical shift values for almost all the protons and carbons in compound **4** except the signal of ethoxy group. Instead, a broad singlet was observed at δ 5.31 which were carefully assigned to the phenolic OH group at position 4 on the benzene ring. Furthermore, the positions of various groups were confirmed by the ^3^ *J*_H-C_ and ^2^ *J*_H-C_ HMBC correlations (Fig. 4). Through the analysis and comparison of the data with the similar known benzoate ester [26] the structure of compound **5** was assigned as *neopentyl-4-hydroxy-3, 5-bis [3-methyl-2-butenyl] benzoate*.

In a bioassay-guided search for acetylcholinesterase (AChE) and butyrylcholinesterase (BChE) inhibitors from medicinal plants, it is interested in this study to identify AChE and BChE inhibiting small molecules from herbal medicinal plants. Compounds **1–5** from *L. quinquelocularis* were tested against AChE and BChE, which represent the most attractive target for drug design and discovery of mechanism-based inhibitors for the treatment of neurone degenerative disorders such as Alzheimer's disease [27]. The percentage of inhibition was first determined at 0.1 mM. The compounds which had enzyme inhibition greater than 50% were subsequently assayed for IC_50_ (50% inhibitory effect) determination. Among the isolated compounds, **3** and **5** showed most effective inhibition activity against AChE and BChE as compared to the standard drugs; allanzanthane and galanthamine in a dose dependent manner. The IC_50_ values of compounds **3** and **5** against AChE were determined to be 1.65 and 3.43 µM, while against BChE, were measured as 5.98 and 9.84 µM respectively. The compounds **2** and **4** showed weak inhibition profile against AChE and BChE (Table 3).

**Table 3:** AChE and BChE inhibitory activities of compound **1**–**5** from *L. quinquelocularis* (IC_50_, µM).

S.No	Compounds	AChE ± SEM^a^	BChE ± SEM^a^
1	**1**	Nil	Nil
2	**2**	8.74±0.07	20.12±0.079
3	**3**	1.65±0.03	5.98±0.079
4	**4**	5.27±0.04	14.76±0.087
5	**5**	3.43±0.02	9.84±0.037
6	Allanzanthane b	2.94±0.45	12.96±0.053
7	Galanthamine b	1.79±0.061	7.98±0.01

a Standard error of mean of five assays.

b Positive control used in the assays.

Note: Data shown are values from triplicate experiments.

## Experimental

### General Experimental Procedure

The melting point was determined by using Kofler hot-stage apparatus (Reichert, Vienna, Austria). Aluminium TLC plates (20×20, 0.5 mm thick) pre-coated with silica gel 60 F_254_ (0.2 mm layer thickness; E. Merck, Darmstadt, Germany) were used for TLC to check the purity of the compounds. Column chromatography (CC) was carried out using silica gel of 230–400 mesh (E. Merck, Darmstadt, Germany). Preparative TLC Glass plates (20×20, 2 mm thick) pre-coated with silica gel 60 F_254_ (0.5 mm layer thickness; E. Merck, Germany) were used for the purification of semi pure compounds. Ceric sulphate and potassium permanganate solutions were used as visualization reagents. The UV spectra (λ_max_ nm) were recorded on Shimadzu UV-2700 spectrophotometer (Shimadzu, Japan) in ethanol. Mass Spectra was recorded on Bruker TOF Mass spectrometers (Billerica, USA) using electrospray ionisation (ESI). The ^1^H NMR and ^13^C NMR spectra were recorded on a Bruker DPX-400 NMR spectrometer (Billerica, USA) (400 MHz for ^1^H and 100 MHz for ^13^C-NMR), using CDCl_3_ as solvents. Further assignments were made by DEPT, COSY, HMQC and HMBC experiments.

### Plant Material

The whole plant of *Lonicera quinquelocularis* was collected from Bara Galli, Hazara division, District Mansehra, in June 2009. It was identified by Professor Dr. Manzoor Ahmad, Plant Taxonomist, Department of Botany, Government Degree College Abbotabad, Pakistan. As the field from where the plant has been collected was open, no authority was responsible to issue permission and the study did not involve endangered and protected species. Likewise there was no restriction from land owner on usability of this specie and the permission was given from the land owner for the collection of this specie. After collection, the voucher specimen has been deposited in the herbarium of the Department of Botany, Government Degree College Abbottabad, Pakistan where a voucher specimen has been deposited in herbarium (Accession No. C-0013).

### Extraction and Isolation

The shade dried whole plant of *L. quinquelocularis* (13 kg) was ground and extracted with ethanol at room temperature (3X25 L). The combined ethanolic extract was evaporated under reduced pressure to obtain a thick greenish gummy material (crude). It was suspended in water and was successively partitioned into various soluble fractions of *n*-hexane (151 g), chloroform (147 g), ethyl acetate (109 g), and n-butanol (53 g) with suitable solvents respectively.

The ethyl acetate soluble fraction was subjected to column chromatography over silica gel (70–230 mesh) eluting with *n*-hexane (100%), *n*-hexane: EtOAc (1: 19–19:1), EtOAc (100%), EtOAc:MeOH (1: 19–19:1), MeOH (100%), in increasing order of polarity to obtain 13 fractions A-M.

Fraction C (4 g) was again chromatographed over silica gel, eluting with *n*-hexane (100%), *n*-hexane: EtOAc (5% hexane/EtOAC to 95% hexane/EtOAc) and EtOAc (100%) to obtain C1–C6 sub fractions. The sub fraction C2 was re-chromatographed over silica gel CC eluting with *n*-hexane (pure), *n*-hexane: EtOAc (5% *n*-hexane/EtOAc to 50% *n*-hexane/EtOAc). From the elution with 5% *n*-hexane/EtOAc colorless oil was obtained which has been identified as a compound **1**. The eluate obtained from 10% n-hexane/EtOAc showed two spots on the TLC which were separated by preparative TLC using n-hexane: EtOAc (4: 1) as solvent system to separate compounds **2** and **3** respectively.

The fraction D (4 g) was re-chromatographed over silica gel eluting with *n*-hexane (100%), *n*-hexane: EtOAc (5% EtOAc/n-hexane to 95% EtOAc/n-hexane) and EtOAc (100%) to obtain D1–D5 sub fractions. The sub fraction D3 was re-chromatographed over silica gel CC eluting with *n*-hexane (pure), *n*-hexane: EtOAc (5% EtOAc/*n*-hexane to 70% EtOAc/*n*-hexane) to give 4 fractions (I–IV). Fraction III was further subjected to preparative TLC eluted with *n*-hexane: CH_2_Cl_2_ (2∶3) for separation of compounds **4** and **5** respectively.

- *Bis (7-acetoxy-2-ethyl-5-methylheptyl) phthalate* (**3**): Colourless oil; UV (MeOH) _max_ nm (log ε): 296 (4.26) and 232 (5.18); IR (dry film) ν_max_ cm^−1^: 1725 (Ester -C = O), 1610 (aromatic C = C) and 1130–1200 (C–O); HREIMS-TOF m/z 562.7642 [M+H]^+^ (calcd. for C_32_H_50_O_8_, 562.7642). ^1^H NMR (400 MHz, CDCl_3_) δ (ppm), ^13^C NMR (100 MHz, CDCl_3_) spectral data and HMBC correlations given in Table 1.
- *Neopentyl-4-ethoxy-3,5-bis [3-methyl-2-butenyl] benzoate* (**4**): Amorphous powder; UV (EtOH) _max_ (log ε): 210 (4.3), 260 (4.5) nm; IR (KBr) ν _max_: 2940, 1705, 1600, 1440, 1300, 1230, 1010, 780 cm^−1^; HREIMS-TOF *m/z* [M]^+^ 372.3742 (calcd for C_24_H_36_O_3_, 372.2156). ^1^H-NMR (CDCl_3_, 400 MHz) δ (ppm), ^13^C-NMR (CDCl_3_, 100 MHz) and HMBC correlations given in Table 2.
- *Neopentyl-4-hydroxy-3,5-bis [3-methyl-2-butenyl] benzoate* (**5**): Amorphous white solid; UV (EtOH) _max_ (log ε): 217 (4.2), 265 (4.5), 295 (4.3) nm; IR (KBr) ν_max_: 2940, 1705, 1600, 1440, 1300, 1230, 1010, 780 cm^−1^; HREIMS-TOF *m/z* [M]^+^ 344.6403 (calcd. for C_22_H_32_O_3_, 344.2549). ^1^H-NMR (CDCl_3_, 400 MHz) δ (ppm), ^13^C NMR (CDCl_3_, 100 MHz) and HMBC correlations given in Table 2;

### Cholinesterase inhibition assay and determination of IC50 values

Acetylcholinesterase (EC 3.1.1.7), butyrylcholinesterase (horse-serum E.C 3.1.1.8), acetylthiocholine iodide, butyrylthiocholine chloride, galanthamine and DTNB (5, 5′-dithiobis [2-nitrobenzoic-acid]) were purchased from Sigma, Pakistan. All other chemicals were of analytical grade and were used as such without further purification. Acetylcholinesterase (AChE) and butyrylcholinesterase (BChE) inhibiting activities were measured according to a modified spectrophotometric method used by Ellman et al. [28]. Protocol and assay conditions were the same as described by Rocha et al. [29].

The Acetylthiocholine iodide and butyrylthiocholine chloride were used as substrates for investigation of acetylcholinesterase and butyrylcholinesterase assays, respectively. The 5,5′-Dithiobis[2-nitrobenzoic-acid] (DTNB) was used for the measurement of cholinesterase activity. The 0.2 mM DTNB in 62 mM sodium phosphate buffer (pH 8.0, 880 µL), test compound solution (40 µL) and acetylcholinesterase or butyrylcholinesterase solution (40 µL) were mixed and incubated for 15 minutes (25°C). The reaction was then initiated by the addition of acetylthiocholine or butyrylthiocholine (40 µL), respectively. The hydrolysis of acetylthiocholine and butyrylthiocholine were monitored by the formation of yellow 5-thio-2-nitrobenzoate anion as a result of the reaction of DTNB with thiocholine, released by the enzymatic hydrolysis of acetylthiocholine and butyrylthiocholine, respectively, at a wavelength of 412 nm (15 min). All the reactions were performed in triplicate using a BMS spectrophotometer (USA). The concentrations of test compounds that inhibited the hydrolysis of substrates (acetylthiocholine and butyrylthiocholine) up to 50% (IC_50_) were determined by monitoring the effect of increasing concentrations of these compounds in the assays on the inhibition values. The final concentration of DMSO in the reaction mixture was maintained at 6%.

## Conclusion

The phytochemical studies of *Lonicera quinquelocularis*; medicinal plant were carried out using latest chromatographic and spectroscopic techniques. Three new and two known compounds were obtained from the ethyl acetate soluble fraction in which compound **3** and **5** showed pronounced cholinesterase inhibition activities with IC_50_ 1.65 and 3.43 µM against AChE and 5.98 and 9.84 µM against BChE respectively in dose dependent manner using reference drugs. The compounds **2** and **4** showed weak inhibition profile while the compound **1** was found inactive for inhibition activity. The studies also showed the structure activity relationship between the new structural features and the corresponding enzymes activities. *Lonicera quinquelocularis* is one of the ingredient of the traditional medicine in some part of the world. Therefore, furthure investigation on this medicnal plant is recommended to exploit its hidden medicinal values.

## Supporting Information

File S1 **Figures S1–S5.** Figure S1a. ^1^H NMR spectra of compound **1.** Figure S1b. ^13^C NMR spectra of compound **1.** Figure S1c. COSEY correlation of compound **1.** Figure S1d. Mass spectra of compound **1.** Figure S2a. ^1^H NMR spectra of compound **2.** Figure S2b. ^13^C NMR spectra of compound **2.** Figure S2c. COSEY correlation of compound **2.** Figure S2d. Mass spectrum of compound **2.** Figure S3a.^1^H NMR spectra of compound **3.** Figure S3b. ^13^C NMR spectra of compound **3.** Figure S3c. COSEY correlation of compound **3.** Figure S3d. HMBC correlation of compound **3.** Figure S3e. Mass spectra of compound **3.** Figure S4a. ^1^H NMR spectra of compound **4.** Figure S4b.^13^C NMR data of compound **4.** Figure S4c. Mass spec data of compound **4.** Figure S4d. HMBC spectra of compound **4.** Figure S5a. ^1^H NMR spectra of compound **5.** Figure S5b. ^13^C NMR data of compound **5.** Figure S5c. Mass spec data of compound **5.**
(DOCX)
