# Supplementary material for: New Cholinesterase Inhibitory Constituents from Lonicera quinquelocularis
Source: PLoS One. 2014 Apr 14;9(4):e94952. doi: 10.1371/journal.pone.0094952 (PMC3986359; doi:10.1371/journal.pone.0094952)
Supplement: File S1 — Figures S1–S5. Figure S1a. 1H NMR spectra of compound 1. Figure S1b. 13C NMR spectra of compound 1. Figure S1c. COSEY correlation of compound 1. Figure S1d. Mass spectra of compound 1. Figure S2a. 1H NMR spectra of compound 2. Figure S2b. 13C NMR spectra of compound 2. Figure S2c. COSEY correlation of compound 2. Figure S2d. Mass spectrum of compound 2. Figure S3a.1H NMR spectra of compound 3. Figure S3b. 13C NMR spectra of compound 3. Figure S3c. COSEY correlation of compound 3. Figure S3d. HMBC correlation of compound 3. Figure S3e. Mass spectra of compound 3. Figure S4a. 1H NMR spectra of compound 4. Figure S4b.13C NMR data of compound 4. Figure S4c. Mass spec data of compound 4. Figure S4d. HMBC spectra of compound 4. Figure S5a. 1H NMR spectra of compound 5. Figure S5b. 13C NMR data of compound 5. Figure S5c. Mass spec data of compound 5. (DOCX) [file pone.0094952.s001.docx]

**Supporting information**


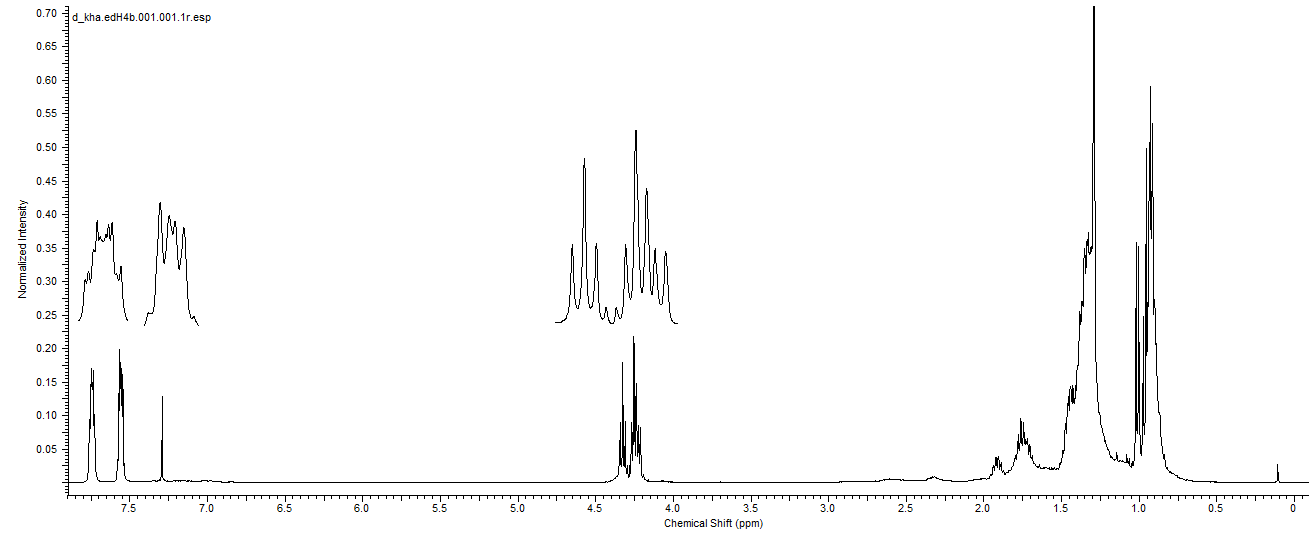


**Fig. S 1a. ^1^H NMR spectra of compound 1**


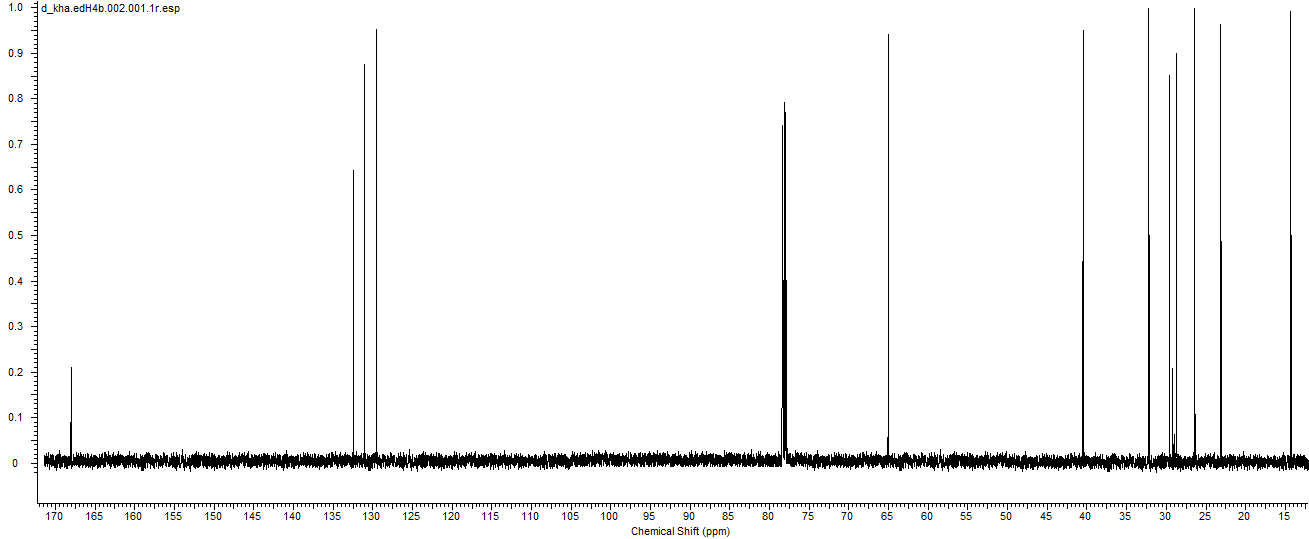


**Fig. S 1b. ^13^C NMR spectra of compound 1**


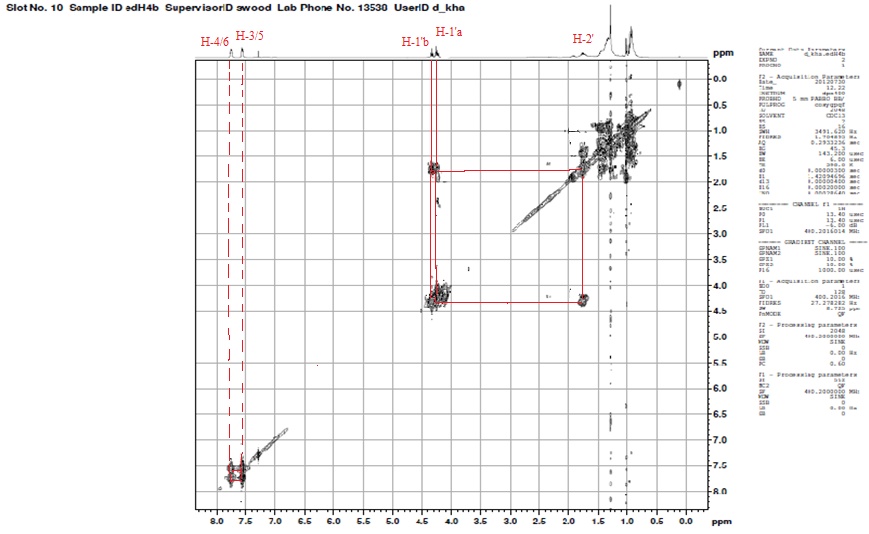


**Fig. S 1c. COSEY correlation of compound 1**


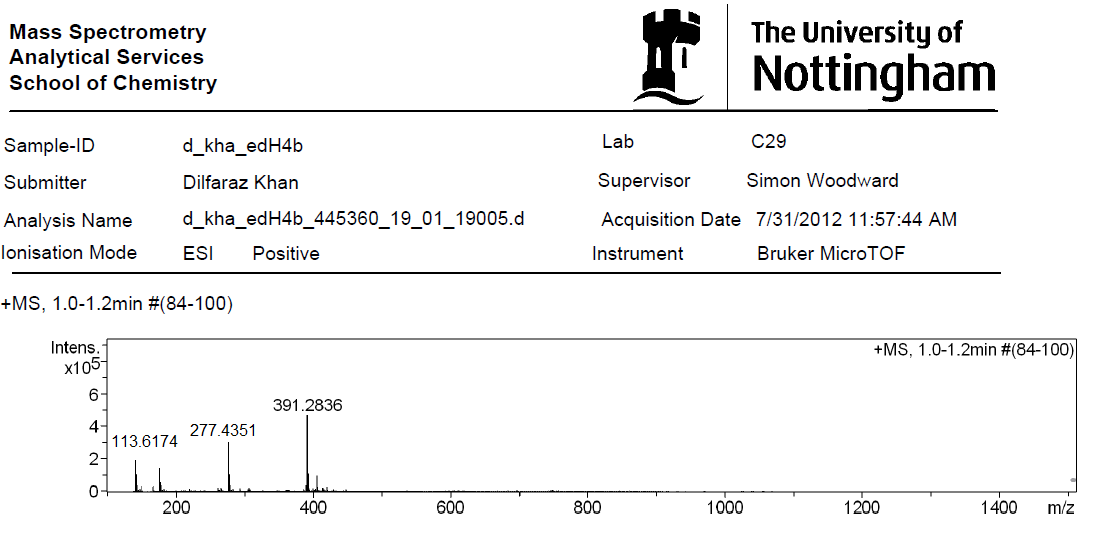


**Fig. S 1d. Mass spectra of compound1**


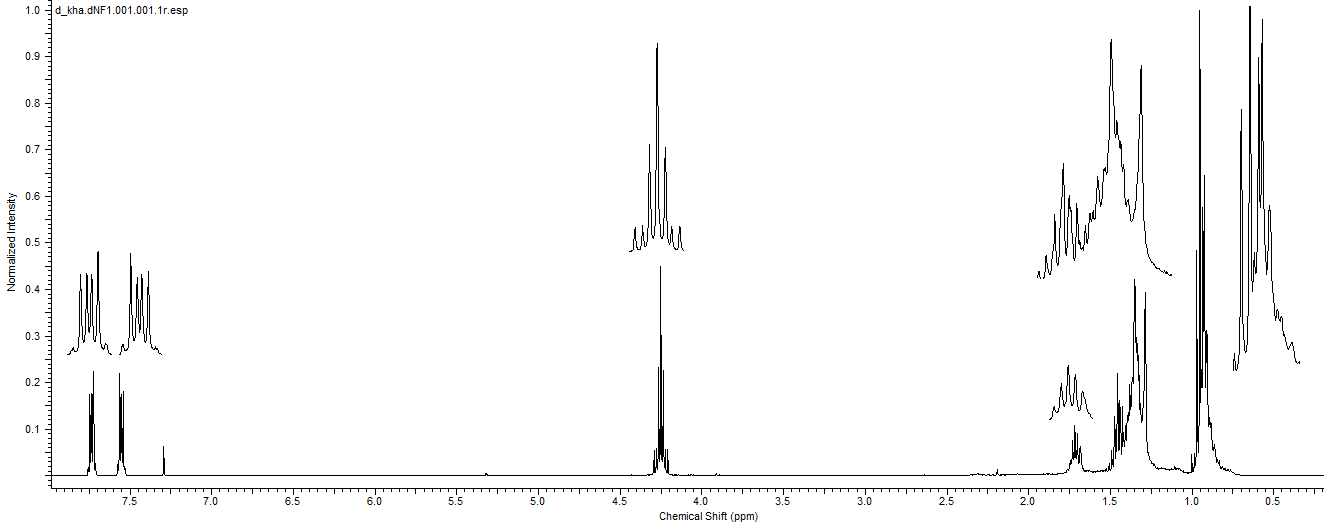


**Fig. S 2a. ^1^H NMR spectra of compound 2**


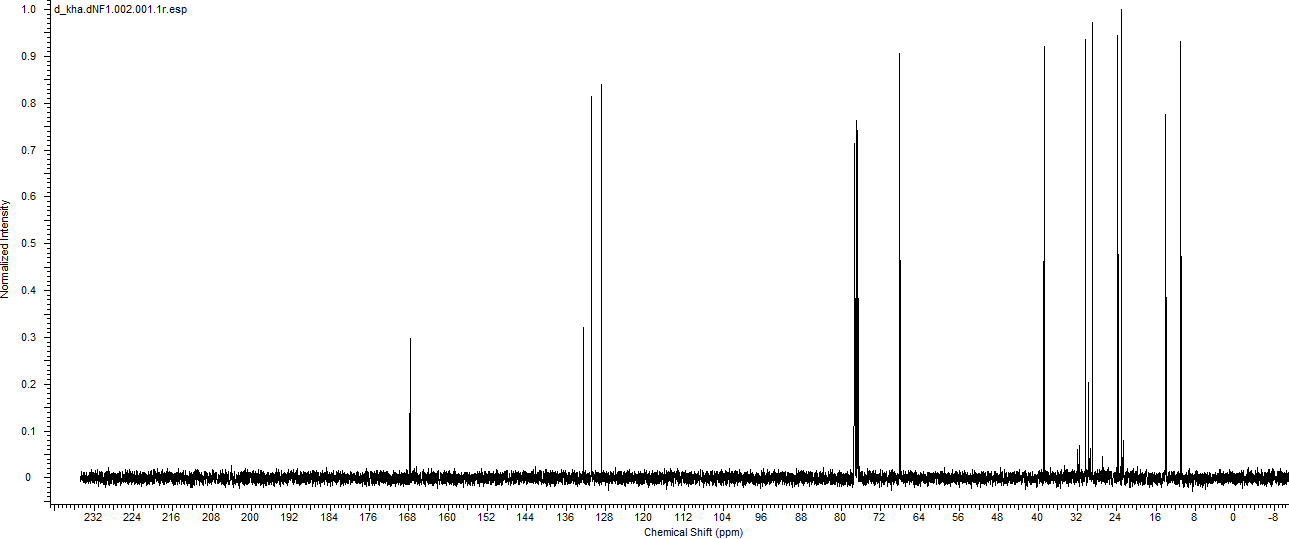


**Fig. S 2b. ^13^C NMR spectra of compound 2**

**
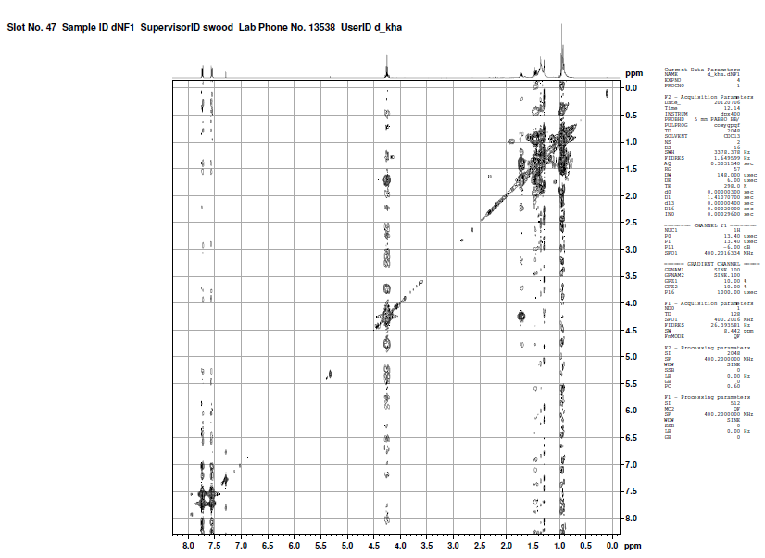
**

**Fig. S 2c. COSEY correlation of compound 2**


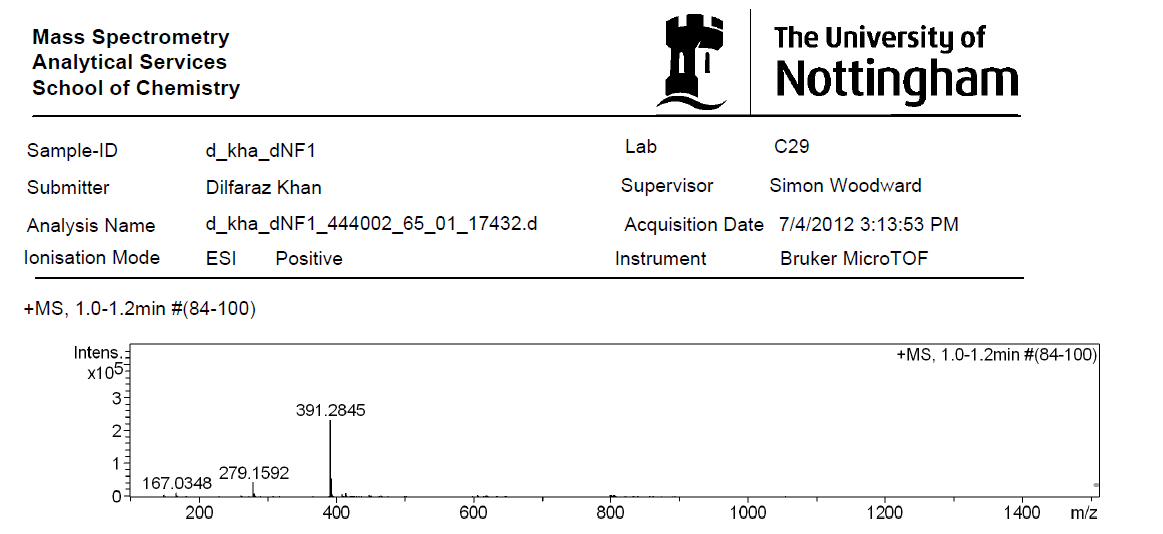


**Fig. S 2d. Mass spectrum of compound 2**

**Fig. S 3a. ^1^H NMR spectra of compound 3**


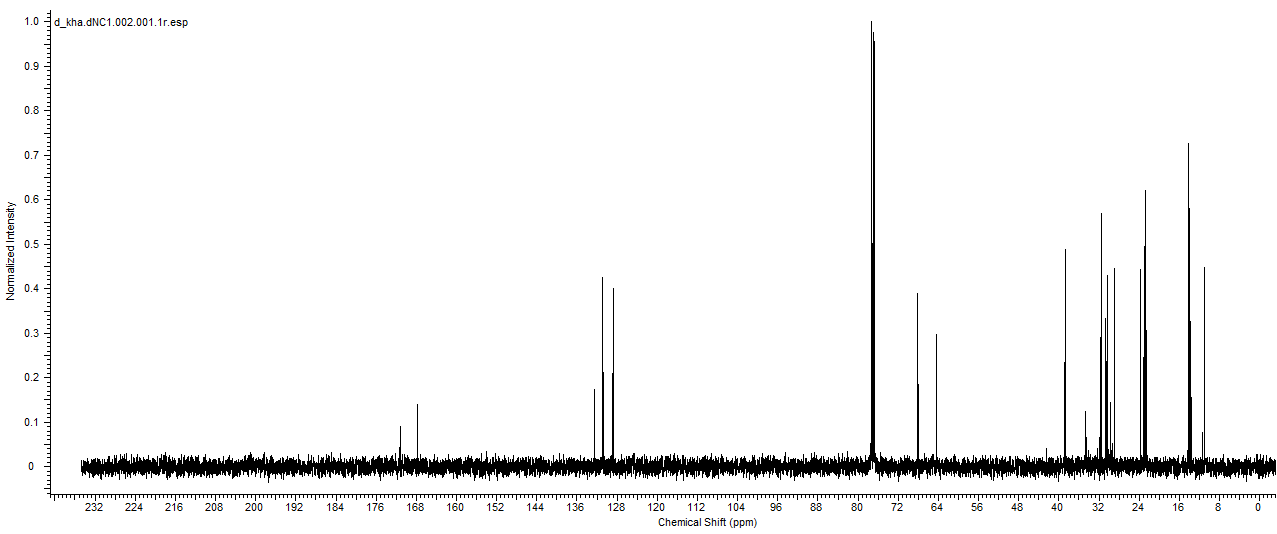


**Fig. S 3b. ^13^C NMR spectra of compound 3**

**
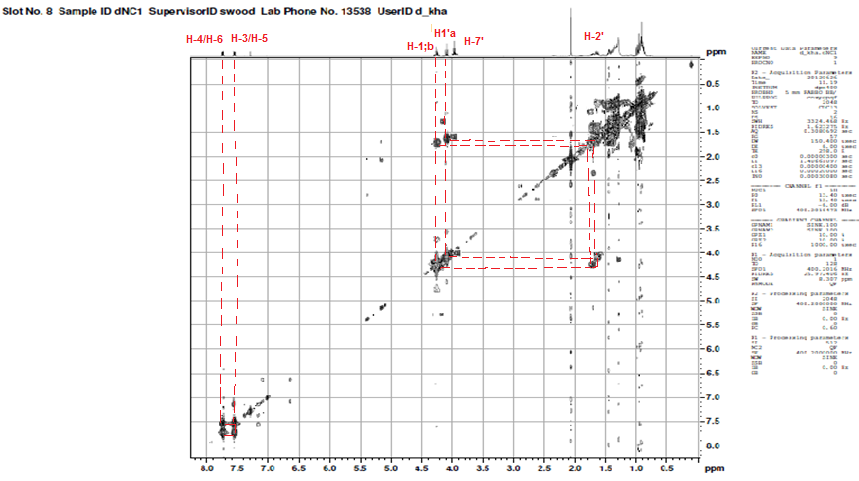
**

**Fig. S 3c. COSEY correlation of compound 3**

**
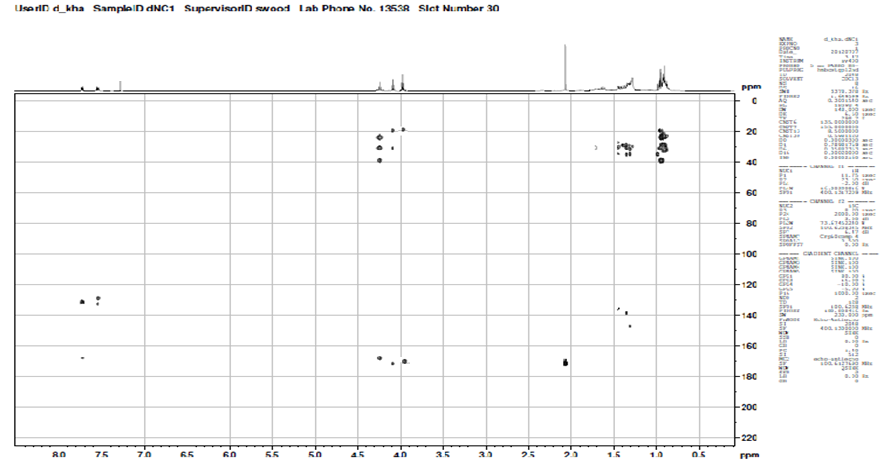
**

**Fig. S 3d. HMBC correlation of compound 3**

**
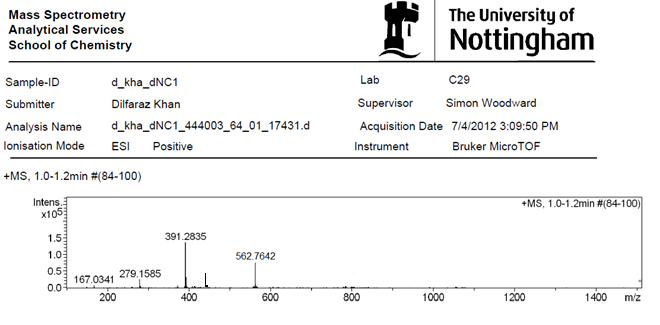
**

**Fig. S 3e. Mass spectra of compound 3**

**Fig. S 4a. ^1^H NMR spectra of compound 4**


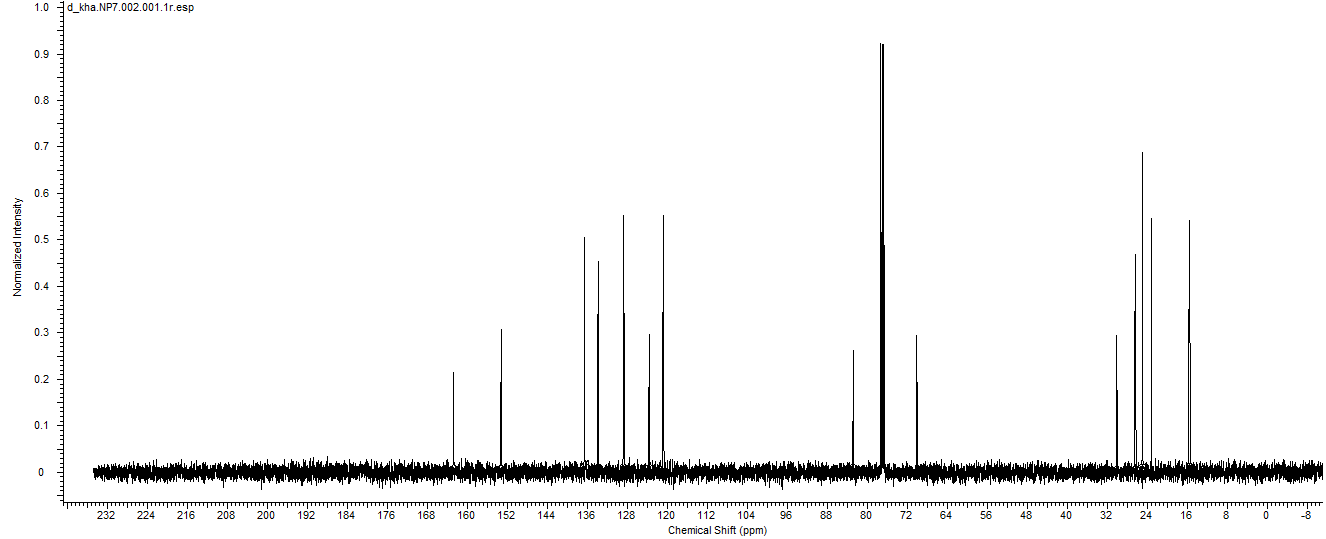


**Fig. S 4b. ^13^C NMR data of compound 4**

**
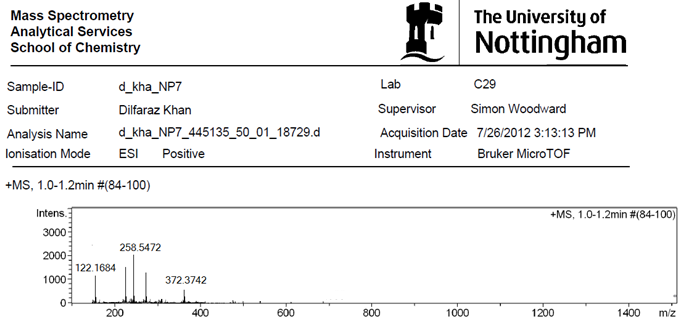
**

**Fig. S 4c. Mass spec data of compound 4**

**
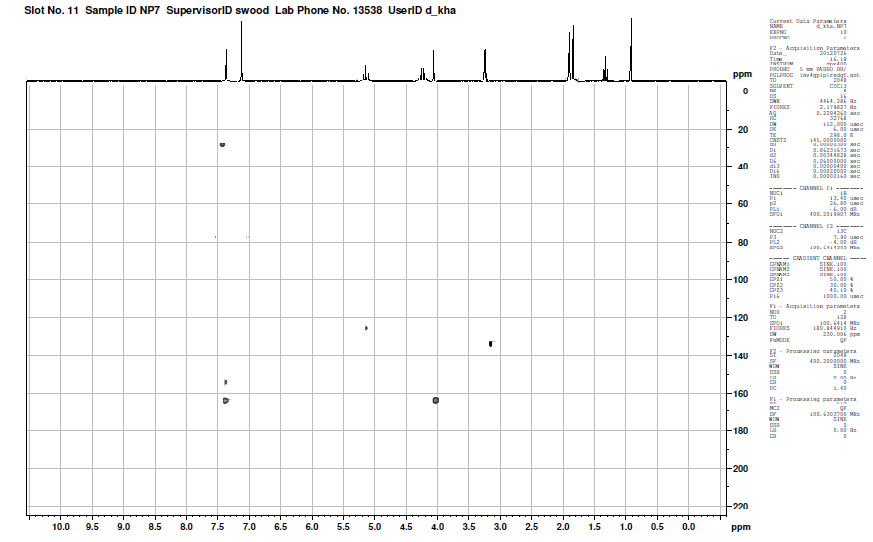
**

**Fig. S 4d. HMBC spectra of compound 4**


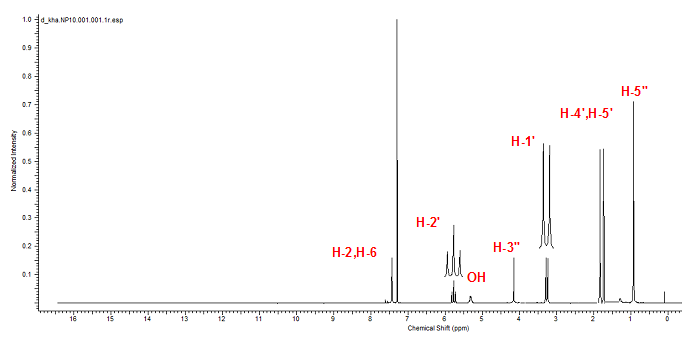


**Fig. S 5a. ^1^H NMR spectra of compound 5**


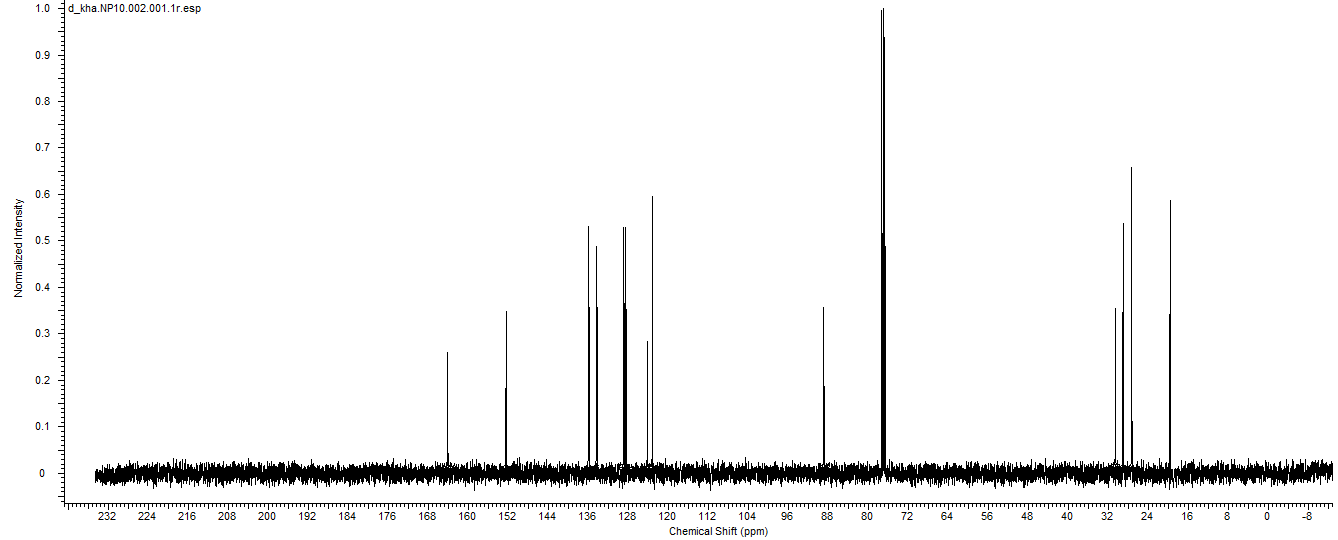


**Fig. S 5b. ^13^C NMR data of compound 5**

**
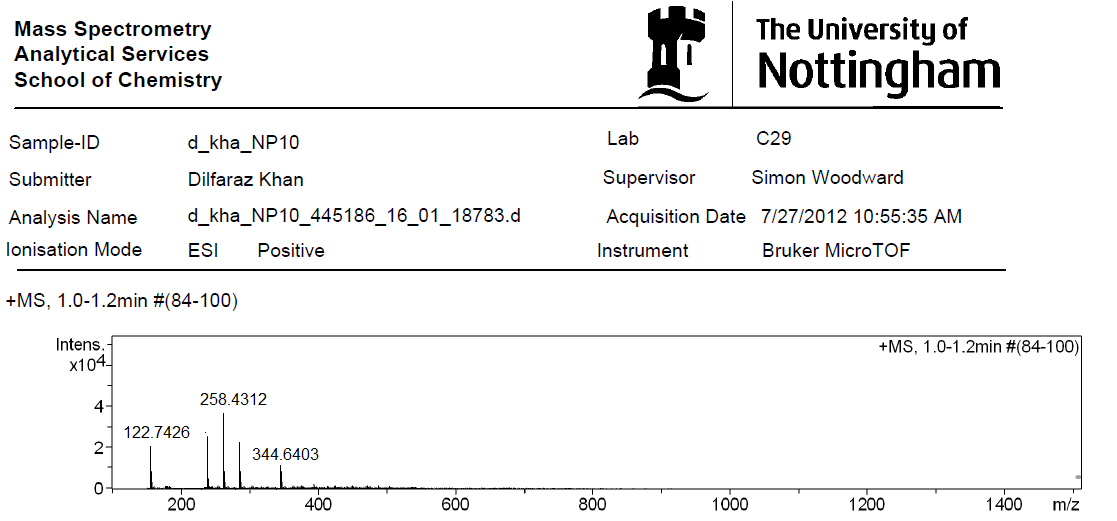
**

**Fig. S 5c. Mass spec data of compound 5**
